# Special Issue “Latest Review Papers in Molecular Oncology 2024”

**DOI:** 10.3390/ijms262411834

**Published:** 2025-12-08

**Authors:** Peter J. K. Kuppen, Carmine Stolfi

**Affiliations:** 1 Department of Surgery, Leiden University Medical Centre, 2300 RC Leiden, The Netherlands; 2 Department of Systems Medicine, University of Rome “Tor Vergata”, 00133 Rome, Italy; carmine.stolfi@uniroma2.it

## 1. Introduction

The Special Issue “Latest Review Papers in Molecular Oncology 2024” represents a continuation of the 2023 edition within the “Molecular Oncology” section of the *International Journal of Molecular Sciences* (IJMS). We aimed to collect high-quality review papers focusing on the most recent advances and emerging trends molecular oncology. A total of seven papers were included in this Special Issue. In this editorial, we summarise the published works and offer some perspectives on future directions of molecular oncology.

## 2. Progress in Molecular Oncology

The field of molecular oncology is moving forward at a fast pace. The progress in technologies such as omics, spatial profiling, non-coding RNA networks, and many other areas means that reviews remain indispensable for synthesising the rapidly expanding body of literature. The goal of this Special Issue was to provide readers not just with summaries of data obtained in the different areas but also with panoramic assessments of how fundamental molecular insights are translating into smart therapeutic and diagnostic strategies. The decoding of the human genome and the advent of high-throughput sequencing have enabled researchers to identify key genetic and epigenetic alterations driving tumour initiation, progression, and resistance to therapy [1]. Cancer, even within distinct types such as colorectal, lung, or breast cancer, is not a single disease but rather a complex collection of molecularly distinct disorders [2]. This knowledge has paved the way for precision medicine, where treatments are tailored to an individual’s molecular tumour profile [3]. Targeted therapies, such as tyrosine kinase inhibitors and monoclonal antibodies, have significantly improved outcomes in cancers such as chronic myeloid leukaemia and non-small-cell lung cancer [4,5]. Furthermore, advances in immuno-oncology, particularly regarding immune checkpoint inhibitors, have revolutionised treatment across multiple tumour types [6]. Many of these topics are discussed in this Special Issue.

## 3. Themes from the Contributions

The papers published in this special issue, and listed below, nicely illustrate recent progress in molecular oncology and applications derived from obtained knowledge.

The first publication in this Special Issue, by Charlotte Delrue et al. (contribution 1), addresses the use of infrared spectroscopy in gynaecological oncology. This work highlights diagnostic opportunities inherent to differences in molecular composition of malignant versus benign tissue, based on the absorption pattern of proteins, lipids, carbohydrates, and nucleic acids. These spectral signatures can not only distinguish malignant from benign tissue but also provide additional information regarding the cellular changes associated with cancer.

The second review by Elena Kalinina (contribution 2) examines glutathione-dependent pathways in cancer cells and how increased glutathione synthesis, glutathione-S-transferases, and glutathione peroxidases shape tumour development, progression, and drug resistance. This accentuates the growing focus on redox biology and cellular metabolism not only as background phenomena but also as active drivers of oncogenesis and therapy failure. The review also discusses how glutathione inhibitors may provide opportunities for cancer treatment.

The next review, by Megan Wu et al. (contribution 3), focuses on the molecular regulation of cancer metastasis, particularly on PAK2, a serine-threonine kinase and a member of the p21-activated kinase (PAK) family. Recent discoveries about the functions of PAK2 in various types of cancers are discussed. The authors hypothesise that a better understanding of the mechanisms behind the different functions of PAK2 will facilitate future development of cancer therapies.

The fourth review, by Milica Nedeljković et al. (contribution 4), addresses triple negative breast cancer (TNBC). The article summarises the molecular subtypes of TNBC and discusses how targets can be derived for tailored treatment strategies instead of conventional drugs, without a molecular basis. Several novel targets and agents are suggested.

The fifth review, by Pablo Pérez-Moreno et al. (contribution 5), explores the molecular interplay between non-coding RNAs and connexins in cancer. By bringing together membrane channel biology, intercellular communication, and regulation of gene expression, this contribution emphasises the crossroads where signalling, cell–cell coupling, and non-coding RNA regulation intersect.

In the penultimate review, by Shosuke Kawanishi et al. (contribution 6), the authors propose a vicious cycle of DNA damage and inflammation in cancer development and progression. They state that inflammatory processes generate reactive oxygen and nitrogen species in both inflammatory and epithelial cells, leading to the induction of oxidative and nitrative DNA damage. In this way, a self-perpetuating loop is created: tissue damage caused by tumour growth induces inflammation, which in turn leads to the accumulation of DNA mutations.

The final review by Jian Zhan and Manfred Jücker (contribution 7), discusses molecular pathways involved in tumour cell resistance to radiotherapy. The authors note that knowledge of such pathways may guide clinical treatment strategies and provide new approaches to overcoming resistance to radiotherapy.

## 4. Concluding Remarks

These reviews illustrate the importance and broad coverage of molecular oncology. It is a vast landscape, from epigenetics, metabolism, immunology, and cell signalling to diagnostics and therapy. The field continues to evolve rapidly, driven by advances in multi-omics technologies, artificial intelligence, accumulating knowledge, and real-world clinical trials. Understanding the underlying molecular mechanisms of cancer development can be translated into improved cancer treatment [7,8,9]. The reviews also demonstrate that apparently similar subtypes, for instance TNBC, can be further subdivided based on molecular characteristics. Therefore, the future approach to cancer treatment will increasingly rely on personalised diagnostics and therapy [10]. Collectively, these reviews show that the field is shifting from a descriptive to an integrative and translational stage. The focus on molecular oncology encourages researchers to move beyond “pathway X is dysregulated” toward “what does this mean for patient stratification, therapy design, and resistance mechanisms?”. That transition remains one of the major challenges in cancer research, but it has clearly been set in motion.
